# Induction of vitiligo after imiquimod treatment of condylomata acuminata

**DOI:** 10.1186/1471-2334-14-329

**Published:** 2014-06-13

**Authors:** Wenfei Li, Hongyan Xin, Lingzhi Ge, Haiyan Song, Wang Cao

**Affiliations:** 1 Department of Dermatology, Qianfoshan Hospital, Shandong University, 16766# Jing-Shi Road, Jinan 250014, China; 2 Shandong Provincial Chest Hospital, Jinan 250013, China; 3 Taishan Medical college, Tai’an 271000, China; 4 Department of Dermatology, Jinan city central Hospital, Jinan 250013, China; 5 Department of Dermatology, Jinan Sixth People’s Hospital, Jinan 250200, China

**Keywords:** Condylomata acuminata, Imiquimod, Vitiligo

## Abstract

**Background:**

Condylomata acuminata (genital warts) is the most common sexually transmitted disease, and imiquimod is the sole FDA-approved medication for combating this condition. Vitiligo associated with imiquimod treatment of condylomata acuminata is rare.

**Case presentation:**

A 28-year-old male with condylomata acuminata of the penis presented to our clinic. After removing his condylomata acuminata, we advised him to use imiquimod 5% cream to prevent relapse. When he presented to our clinic again about 12 weeks later, he complained of vitiligo patches on his penis and scrotum. Physical examination showed vitiligo patches involving the glans penis, shaft of the penis, and scrotum, and remaining pigmented areas within the plaques of vitiligo.

A skin biopsy of the dorsal surface of the penis showed a complete absence of melanocytes and melanin granules in the basal layer; the dermis was normal.

**Conclusion:**

This is the first report of a case of imiquimod-induced vitiligo diagnosed by histopathological examination. This adverse effect should be considered when dermatologists prescribe this medication.

## Background

Condylomata acuminata (genital warts) is the most common sexually transmitted disease. It is caused by human papilloma virus (HPV) infection, which may contribute to cervical cancer [1]. The primary goals of treatment are removal of the visible warts and prevention of recurrence. The many methods in the therapy of condylomata acuminata include cryotherapy, electrodessication, CO_2_ laser, trichloroacetic acid, podophyllin resin 10%–25%, and imiquimod 5% cream [2,3]. Among them, imiquimod is the sole FDA-approved medication for combating condylomata acuminata. It is also used to treat certain diseases of the skin such as Bowen’s disease, common and plantar warts, molluscum contagiosum, herpes simplex, Paget’s disease, basal cell carcinoma, and superficial squamous cell carcinoma. Although regarded as a safe drug, mild-to-moderate, local and systemic, adverse effects of imiquimod may occasionally occur [4]. Since vitiligo-like hypopigmentation associated with imiquimod treatment of condylomata acuminata was first reported by Brown in 2005, to the best of our knowledge there have been only eight patients with either vitiligo or vitiligo-like hypopigmentation associated with imiquimod treatment of condylomata acuminata described in the literature [5-11]. The clinical features of these patients are listed in Table 1.

**Table 1:** Comparison of published cases of imquimod-induced vitiligo or vitiligo-like depigmentation in English literature

**Case**	**Author (year)**	**Age (year)/ gender**	**Diagnosis**	**Site**	**Clinical presentation**	**Pathology**	**Family genetic history**
1	Brown et al. 2005 [5]	25/M	Vitiligo-like hypopigmentation	Scrotum	Multiple depigmented patches on the scrotum ranging from 3 mm to 2 cm	No	No report
2	Stefanaki et al. 2006 [6]	32/M	Vitiligo	Dorsal surface of penis, scrotum, and pubic area	Vitiligo, slight repigmentation	No	Yes
3	Senel et al. 2007 [8]	32 M	Vitiligo-like depigmentation	Glans penis, shaft of penis, and scrotum	Depigmented areas	No	No
4	Al-Dujaili et al. 2007 [7]	21/M	Vitiligo	Penile shaft and scrotum	Depigmented patches	No	No report
5	Serrão et al. 2008 [9]	26/M	Vitiligo-like depigmentation	Shaft of penis	Vitiligo-like depigmentation	No	No report
6	Zhang et al. 2011 [10]	25/M	Vitiligo	Coronoid sulcus and corpus penis	Ivory-white patch nearly 4 × 2 cm	No	No report
7	Zhang et al. 2011 [10]	22/M	Vitiligo	Penis	Depigmented patches with irregular pigmented edges	No	No report
8	Wang et al. 2013 [11]	36/F	Vitiligo	Perineum and perianal	Depigmentation patches with clear demarcation lines	No	No

Here, we present an unusual case of imiquimod-induced vitiligo in a 28-year-old male, whose diagnosis was made using clinical and histopathological methods.

## Case presentation

**Figure 1:**
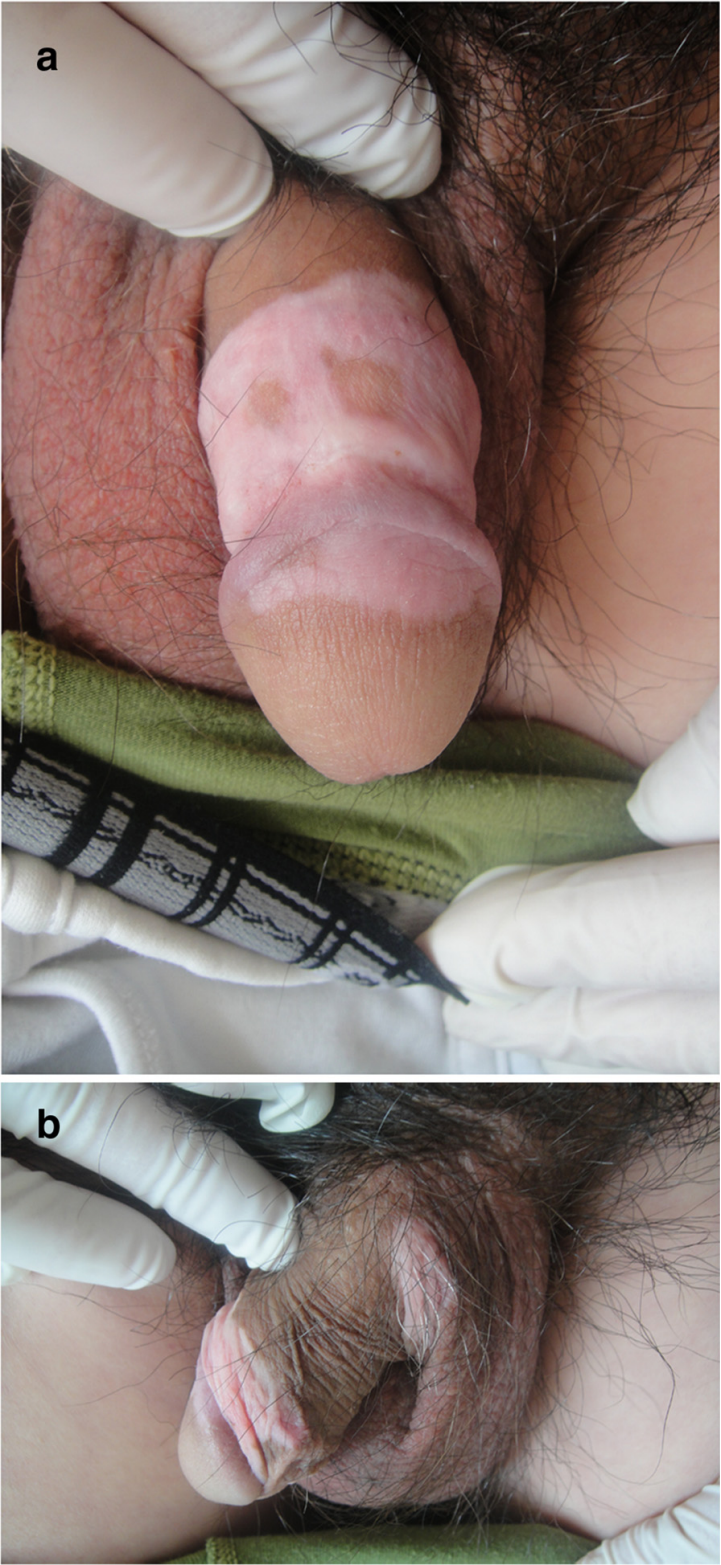
**Vitiligo patches involving the glans penis, shaft of the penis, and scrotum after the use of imiquimod 5%. a**. Vitiligo on the patient’s penis,some remaining pigmented areas within the vitiligo. plaques . **b**. vitiligo on the patient’s scrotum.

A 28-year-old Chinese male presented to our clinic with a 3-year history of condylomata acuminata of the penis. His lesions had been previously treated several times with liquid nitrogen and electrodessication without causing any pigmentary changes, but his problem had relapsed half a month before presentation and now he was presenting with five new warts. The patient was treated with electrodessication to remove his condylomata acuminata. When his wound healed 12 days later, he was advised to use imiquimod 5% cream for relapse prevention. He applied the cream for three nights weekly and washed it off in the morning. Before long, he noticed some irritation from erythema and excoriation in the treated areas, but he persisted in the application. After about 12 weeks of continuous use, he again presented to the clinic complaining of vitiligo-like depigmentation of the macules on his penis. He was instructed to stop using the imiquimod, but the macules in the treated areas gradually enlarged and asymptomatically merged; a vitiligo-like, depigmented patch also appeared on his scrotum during the following 10 days. After 4 weeks of applying tacrolimus 0.1% ointment, the lesions became slightly repigmented, and the area of vitiligo lesions stabilized. Unfortunately, no further repigmentation occurred after 3 months of follow-up.Laboratory examination and a skin biopsy were recommended. He and his family members had no history of vitiligo, other depigmented dermatoses, or autoimmune disorders. He denied use of any other topical treatment. Physical examination showed vitiligo patches involving the glans penis, the shaft of the penis, and the scrotum, along with some remaining pigmented areas within the vitiligo plaques (Figure 1). Wood’s light accentuated the depigmented areas. He was not tired or irritable. EKG, chest X-ray, and thyroid and abdominal ultrasonic scans indicated no cardiac, pulmonary, bilateral thyroid, hepatic, splenic, nephritic, or other organ involvement.

**Figure 2:**
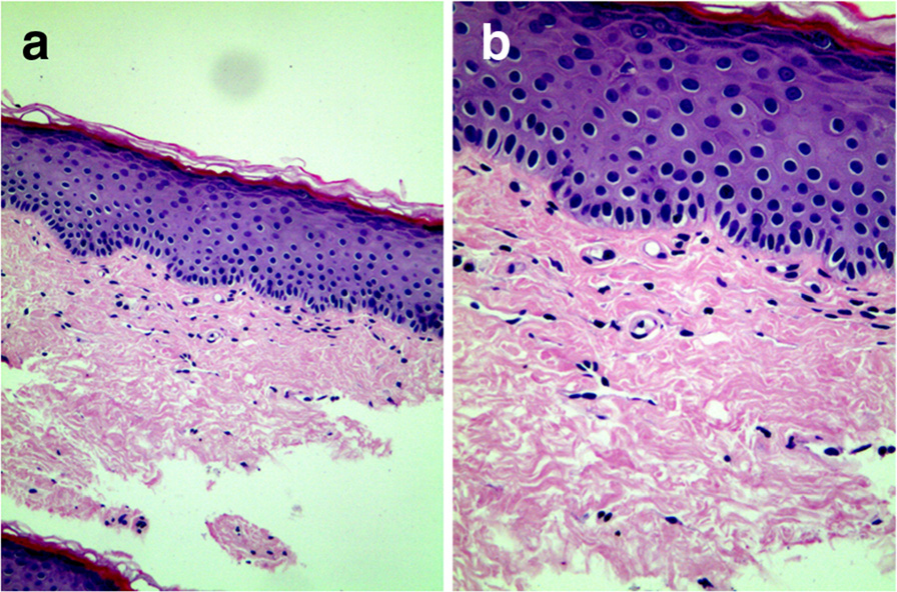
**Loss of melanocytes and melanin granules. a**. Normal stratum corneum, stratum granulosum, and stratum spinosum, with loss of melanocytes and melanin granules in the basal layer. Dermis showing no abnormalities (H&E ×100). **b**. loss of melanocytes and melanin granules in the basal layer (H&E ×200).

**Figure 3:**
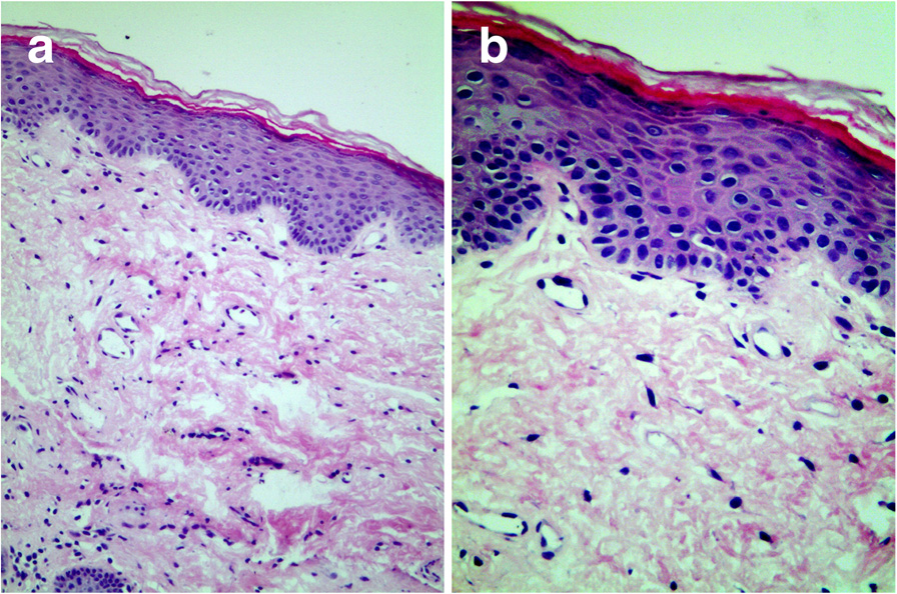
**Absence of melanin granules. a**. Absence of melanin granules in epidermis. Normal dermis. (*Masson-Fontana* stain for melanin ×100). **b**. Absence of melanin granules in epidermal basal cell layer. *(Masson-Fontana* stain for melanin ×200).

**Figure 4:**
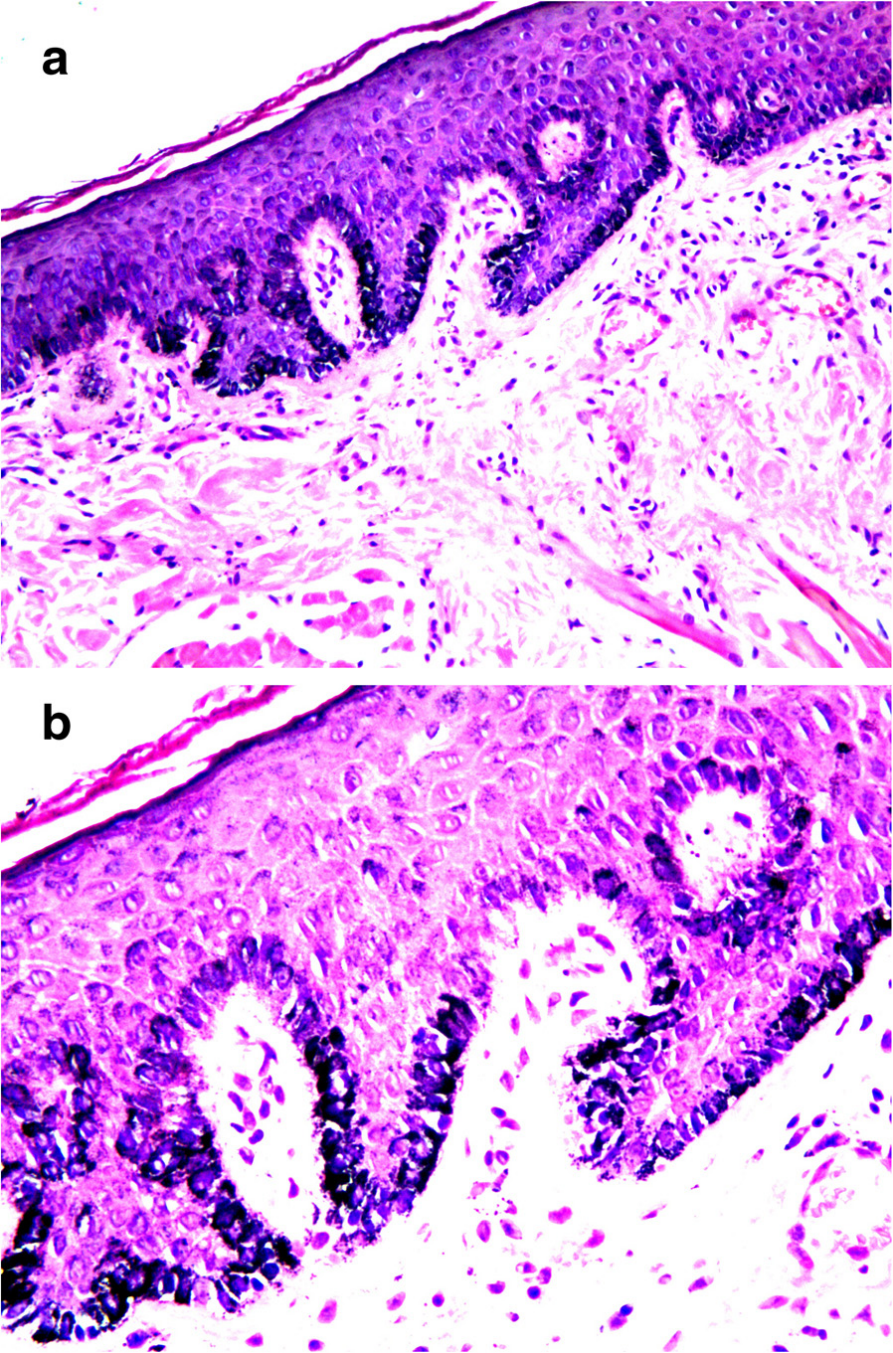
**Normal skin (control): normal of melanocytes and melanin granules. a**. presence of melanocytes and melanin granules in epidermis. Normal dermis *(Masson-Fontana* stain for melanin ×100). **b**. presence of melanocytes and melanin granules in epidermal basal cell layer. *(Masson-Fontana* stain for melanin ×200).

Additional laboratory analysis revealed normal counts of white blood cells, red blood cells and blood platelets; normal elevated erythrocyte sedimentation rate; normal thyroid function tests; negative antistreptolysin O titer and rheumatoid factor; normal serum immunoglobulins G, M, and A, E levels; normal liver enzyme level; normal blood urea nitrogen and creatinine level; normal blood glucose level; negative antinuclear and antineutrophil cytoplasmic antibodies; negative HIV and syphilis antibodies; normal hepatitis A, B, and C serology; normal urine and stool analysis.A skin biopsy was performed on the dorsal surface of the penis, which showed a complete absence of melanocytes and melanin granules in the basal layer but with a normal dermis (Figure 2). Loss of melanocytes and melanin granules in the epidermis was highlighted by Fontana-Masson staining (Figure 3). Melanocytes and melanin granules exist in the epidermal basal-cell layer of normal skin (Figure 4). He was diagnosed, clinically and pathologically, with imiquimod-induced localized vitiligo.

## Discussion

Imiquimod is an immune response modifier with the chemical structure 1-(2-methylpropyl)-1H-imidazo[4,5-c]quinolin-4-amin. Since imiquimod was authorized on the US market in 1997 and on the Chinese market in 2003, it has been approved as a patient-applied topical treatment for condylomata acuminata in adults. We find it is a well-tolerated drug but with the frequent side effects of erythema, burning, blistering, and excoriation. We also noted its other adverse effect of inducing localized vitiligo, which occurred in the treated areas.

Vitiligo is a condition characterized by depigmentation of the skin and mucous membranes, with well-demarcated, depigmented macules and patches. The etiology of vitiligo is unknown, but research suggests that it may involve autoimmune, genetic, and other factors. Autoimmune disorders are often associated with thyroid abnormalities, pernicious anemia, systemic lupus erythematosus, and other diseases. Our patient denied any family history of vitiligo or autoimmune disorder, the possibility of which was excluded by laboratory analysis. He denied use of any other topical treatments in the areas treated with imiquimod 5% cream. The features of his depigmented patches and histopathology support the diagnosis of vitiligo. Therefore, we believe that the vitiligo patches of our patient were induced by the imiquimod. We were able to obtain a small amount of superficial skin from the dorsal surface of the genital lesions in the perineal region. All eight of the patients reported in the English literature refused biopsies of the depigmented areas, and one even refused to have the lesions photographed. Therefore, our patient is the first to undergo histopathological examination and whose diagnosis was based on clinical and histopathological findings.

The possible mechanism for the destruction of the human papilloma virus (HPV) by imiquimod is that it stimulates peripheral blood monocytes, macrophages, and dendritic cells to produce such cytokines as interferon alfa (IFN-α), interleukin-12 (IL-12), and tumor necrosis factor alfa (TNF-α), so imiquimod can enhance the host’s innate and cellular immune response and combat anogenital HPV infection [12,13]. To study the safety and effectiveness of imiquimod 5% cream in the treatment of external anogenital warts, Edwards et al. applied it on 109 patients; 50% of the patients experienced eradication of all treated baseline warts [14]. Other studies also indicated that imiquimod 5% cream was effective in treating condylomata acuminata. However, imiquimod not only kills the HPV but also destroys melanocytes. Similarly, the mechanism of imiquimod-induced vitiligo may be that the medication activates the Langerhans cells in the lesions via antigen presentation, leading to the destruction and apoptosis of the melanocytes. Imiquimod-induced apoptosis of melanocytes was confirmed by TUNEL assay, Hoechst 33258 staining, and measuring mitochondrial membrane potential in melanocytes [15]. Moreover, imiquimod can induce cytokines such as IFN-α, TNF-α, IL-6, IL-8, and nitric oxide to cause vitiligo [16]. Additionally, imiquimod binds to Toll-like receptor-7 and −8, increasing production of proinflammatory cytokines such as IFN-α, TNF-α, and LI-12 [17,18], which play a role in the pathogenesis of vitiligo.

Accompanying the use of imiquimod on increasing numbers of patients with condylomata acuminata, dermatologists should keep this potential side effect in mind.

## Conclusion

Imiquimod 5% cream, as an immune response modifier and a safe drug, is used to treat condylomata acuminata, basal cell carcinoma, Bowen’s disease, common and plantar warts, molluscum contagiosum, and other disorders. However, mild-to-moderate, local and systemic, adverse effects of imiquimod may occasionally occur. Among its adverse effects, imiquimod-induced vitiligo should be anticipated when dermatologists prescribe this drug.

## Consent

Written informed consent was obtained from the patient for publication of this case report and any accompanying images. A copy of the written consent is available for review by the Editor of this journal. This study was proved by Institutional Review Board of Qianfoshan Hospital, Shandong University.

## Competing interests

The authors declare that they have no potential conflicts of interest to disclose.

## Authors’ contributions

Study concept and design: WFL. Acquisition of data: HYX and LZG. Analysis and interpretation of data: HYS and WC. Drafting of the manuscript: HYX and LZG. Critical revision of the manuscript for important intellectual content: WFL. All authors read and approved the final manuscript.

## Pre-publication history

The pre-publication history for this paper can be accessed here:

http://www.biomedcentral.com/1471-2334/14/329/prepub
